# Altered protein secretion in Batten disease

**DOI:** 10.1242/dmm.049152

**Published:** 2021-12-06

**Authors:** Robert J. Huber

**Affiliations:** Department of Biology, Trent University, Life & Health Sciences Building, 1600 West Bank Drive, Peterborough, Ontario K9L 0G2, Canada

**Keywords:** Batten disease, Cerebrospinal fluid, *Dictyostelium discoideum*, Model system, Neuronal ceroid lipofuscinosis, Secretion, Urine

## Abstract

The neuronal ceroid lipofuscinoses (NCLs), collectively known as Batten disease, are a group of neurological diseases that affect all ages and ethnicities worldwide. There are 13 different subtypes of NCL, each caused by a mutation in a distinct gene. The NCLs are characterized by the accumulation of undigestible lipids and proteins in various cell types. This leads to progressive neurodegeneration and clinical symptoms including vision loss, progressive motor and cognitive decline, seizures, and premature death. These diseases have commonly been characterized by lysosomal defects leading to the accumulation of undigestible material but further research on the NCLs suggests that altered protein secretion may also play an important role. This has been strengthened by recent work in biomedical model organisms, including *Dictyostelium discoideum,* mice, and sheep. Research in *D. discoideum* has reported the extracellular localization of some NCL-related proteins and the effects of NCL-related gene loss on protein secretion during unicellular growth and multicellular development. Aberrant protein secretion has also been observed in mammalian models of NCL, which has allowed examination of patient-derived cerebrospinal fluid and urine for potential diagnostic and prognostic biomarkers. Accumulated evidence links seven of the 13 known NCL-related genes to protein secretion, suggesting that altered secretion is a common hallmark of multiple NCL subtypes. This Review highlights the impact of altered protein secretion in the NCLs, identifies potential biomarkers of interest and suggests that future work in this area can provide new therapeutic insight.

## Abstract (precis)

**Summary:** This Review discusses work in different model systems and humans, examining the impact of altered protein secretion in the neuronal ceroid lipofuscinoses group of diseases to provide novel therapeutic insights.

## Introduction

The neuronal ceroid lipofuscinoses (NCLs) are a family of autosomal recessive (Box 1) neurological diseases characterized by the accumulation of undigestible ceroid lipofuscin (Box 1) in various cell types, which results in progressive neurodegeneration (Cooper et al., 2015). Commonly known as Batten disease, the NCLs cause severe clinical symptoms, including vision loss, motor and cognitive decline, seizures, and premature death (Radke et al., 2015). Although Batten disease affects all ages and ethnicities it is recognized as the most common form of neurodegeneration in children. There are 13 different subtypes of NCL that share broadly similar clinical and pathological profiles (Nelvagal et al., 2020) but each subtype is caused by a mutation in a distinct gene (Butz et al., 2020) (Table 1). The NCLs are commonly known as lysosomal storage diseases because of the lysosomal accumulation of ceroid lipofuscin. However, the precise functions of NCL-related genes and proteins, and the molecular and cellular mechanisms underlying the NCLs remain incompletely understood, which has motivated the use of a variety of model systems to provide clues into the biological processes affected by mutations in NCL-related genes (Huber et al., 2020a; Minnis et al., 2020). Early work on patient samples suggested the involvement of altered protein secretion in the NCLs (Table 2). This is consistent with neuronal signaling relying on the regulated secretion of proteins and other molecules (Chung et al., 2016), and aberrant secretion being associated with neurodegeneration (Gonçalves et al., 2016; Pérez et al., 2016).

**Box 1. Glossary**

**14-3-3 protein zeta/delta:** Adaptor protein associated with a variety of signaling pathways such as those regulating apoptosis.

**Angiotensin:** Peptide hormone that regulates blood pressure.

**Astrocyte:** Non-neuronal cell in the nervous system that provides biochemical support for neurons.

**ATG genes:** Genes that encode proteins involved in autophagy.

**Autophagosome:** Double-membrane structure that forms around intracellular material slated for degradation by autophagy.

**Autosomal recessive:** Genetic condition caused by mutations on both copies of a gene in an individual.

**Bcl-2-associated athanogene 2 (BAG2) pathway:** Comprises proteins that interact with B-cell lymphoma 2 (Bcl-2) to prevent cell death.

**Cathepsins:** Family of lysosomal proteases that cleave peptide bonds in proteins. Mutations in genes encoding cathepsin D or F (*CTSD* or *CTSF*, respectively) cause CLN10 or CLN13 disease, respectively.

**Cationic trypsinogen:** Member of the trypsin family of serine proteases, which is secreted by the pancreas.

**CCL5, CCL9, CXCL2:** Secreted proteins that function as chemotactic cytokines.

**Cerebellar granule neuron precursors**: Most abundant neurons in the cerebellum, which are generated from the hindbrain during late embryogenesis.

**Ceroid lipofuscin:** Autofluorescent intracellular accumulations composed of undigestible lipids and proteins.

**CLN1 disease:** Infantile-onset form of NCL caused by mutations in the *PPT1* gene.

**CLN2 disease:** Late infantile-onset form of NCL caused by mutations in the *TPP1* gene.

**CLN3 disease:** Juvenile-onset form of NCL caused by mutations in the *CLN3* gene. Most common NCL subtype.

**CLN4 disease:** Adult-onset form of NCL caused by mutations in the *DNAJC5* gene. Also known as Kufs or Parry disease.

**CLN7 disease:** Late infantile-onset form of NCL caused by mutations in the *MFSD8* gene.

**Conditioned buffer:** Extracellular protein-containing fluid that surrounds submerged *D. discoideum* cells in culture during starvation.

**Conditioned medium:** Extracellular nutrient- and protein-containing fluid that surrounds submerged cells in culture during growth.

**Contractile vacuole (CV)**: Osmoregulatory organelle in *D. discoideum* that also plays a role in ion homeostasis and unconventional protein secretion.

**Dipeptidyl peptidase 4 (DPP4):** Extracellular glycoprotein with serine peptidase activity that plays a role in a variety of cellular processes, including immunity and glucose metabolism.

**Early stages of development (*D. discoideum*):** Period from the onset of starvation to mound formation.

**Gene ontology (GO) term enrichment analysis:** Bioinformatics approach that examines a list of genes to identify enriched localization and functional annotations.

**Glutathione:** Neuroprotective factor in the brain that protects against oxidative stress.

**Golgi reassembly-stacking protein (GrpA):** *D. discoideum* protein that regulates unconventional protein secretion.

**Glycosylation:** Post-translational modification that attaches sugar molecules to proteins.

**Heat shock protein 90:** Chaperone that assists in the folding and stabilization of proteins.

**High endothelial venule protein:** Secreted protein that interacts with the extracellular matrix to facilitate cell adhesion.

**Microglia:** Non-neuronal cells in the nervous system that maintain the health of neurons and function in immune defense.

**Misfolding-associated protein secretion:** An unconventional mechanism of protein secretion that exports misfolded cytosolic proteins outside the cell.

**Multivesicular bodies:** Specialized type of late endosome that contains internal vesicles formed by the invagination of the endosomal membrane.

**STRING:** Online bioinformatics resource that performs functional enrichment analyses and predicts protein-protein interaction networks.

**Tissue factor:** Secreted protein involved in initiating the clotting response and hemostasis.

**Table 1.**
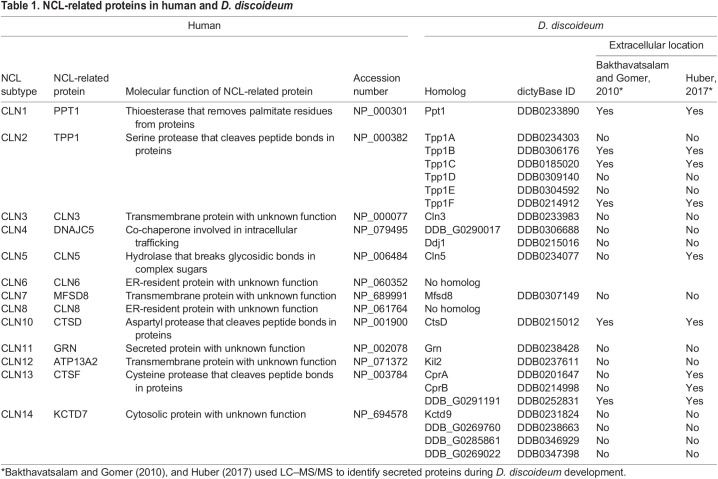
NCL-related proteins in human and *D. discoideum*

**Table 2.**
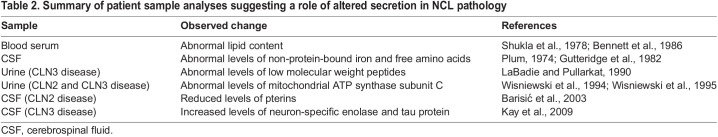
Summary of patient sample analyses suggesting a role of altered secretion in NCL pathology

**Fig. 1.**
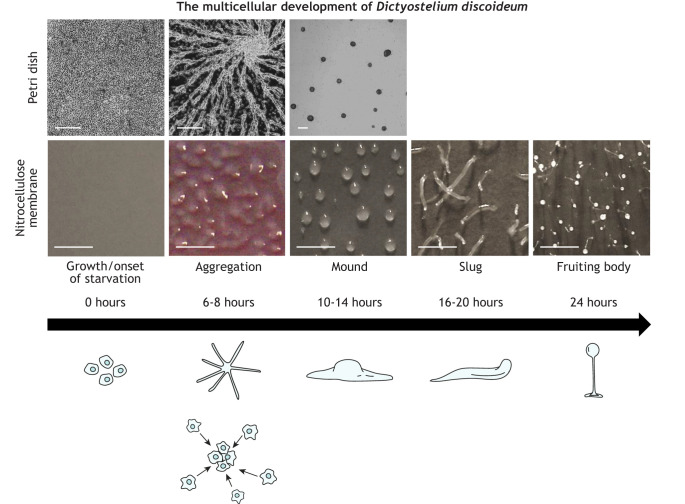
**The life cycle of *Dictyostelium discoideum*.** Microscopy images showing the development of *D. discoideum* cells when applying two commonly used experimental setups, i.e. cells adhered to Petri dishes and submerged in a development buffer (top), and cells adhered to nitrocellulose membranes soaked in development buffer (bottom). During the vegetative, i.e. growth, phase of the life cycle, amoebae internalize their food source using phago- or pinocytosis and undergo mitotic cell division. Upon food depletion (onset of starvation), cells stop dividing and initiate a developmental sequence that begins with secretion of cAMP, which functions as a chemoattractant to stimulate cellular adhesion and the formation of multicellular mounds. A mound then undergoes a series of morphological changes to form a slug that migrates on the substratum in response to light and temperature stimuli. Cells within the slug terminally differentiate into either stalk or spores that form the mature fruiting body. When a food source becomes available, the spores germinate, thereby allowing the cells to restart the life cycle. Notice: Cells submerged in development buffer within Petri dishes do not progress past the mound stage. Scale bars: 250 µm (Petri dishes), 1000 µm (nitrocellulose membrane). A modified version of this figure was previously published in Huber et al. (2021).

This Review describes work in *Dictyostelium discoideum* (Box 2), which highlights the roles of NCL-related proteins in protein secretion (Box 3). Most of the work in this area has focused on the role of *D. discoideum* Cln3, the homolog of human ceroid-lipofuscinosis neuronal 3 (CLN3) (Table 1), in regulating protein secretion during growth and the early stages of development (Box 1) (Fig. 1). However, recent work on other *D. discoideum* NCL-related proteins suggests that altered secretion is a common hallmark of multiple NCL subtypes. This Review also evaluates recent work in mammalian models of NCL, which has examined how altered secretion in the NCLs can provide biomarkers for diagnosis and prognosis, as well as for monitoring therapy response. Overall, the aim of this Review is to highlight how studying aberrant protein secretion in the NCLs can complement existing approaches for tackling the disease, such as understanding lysosomal defects.

**Box 2. *Dictyostelium discoideum* as a model system to study the NCLs**

Many non-mammalian and mammalian models have been used to study the localization and function of NCL-related proteins, providing the foundation for our current understanding of NCL pathology (Huber et al., 2020b). One such organism is the eukaryote *D. discoideum* (McLaren et al., 2019), a soil microbe that has historically served as an excellent model system for studying conserved cellular and developmental processes (Mathavarajah et al., 2017). More recently, *D. discoideum* has been used as a biomedical model to study a variety of human diseases, such as Alzheimer's disease (Sharma et al., 2019), Huntington's disease (Myre et al., 2011) and cancer (Mathavarajah et al., 2021). The 34-megabyte *D. discoideum* genome has been estimated to encode ∼12,500 proteins (Eichinger et al., 2005), including homologs of 11 of the 13 human proteins that are linked to the NCLs (Huber, 2016). Among the many benefits of using *D. discoideum* to study protein function is its 24-h life cycle that comprises single-cell as well as multicellular phases (Mathavarajah et al., 2017) (Fig. 1). Furthermore, cAMP – which was shown to be affected in a CLN3 disease mouse model (Aldrich et al., 2016) – plays a central role in regulating *D. discoideum* development (Kawabe et al., 2019) in a primitive neurotransmission-like scenario. Therein, cAMP serves as the signal transmitter. The significance of studying NCL-related proteins in *D. discoideum* has recently been reviewed (Huber, 2020). Importantly, research in *D. discoideum* has shown that NCL-related proteins have important roles in protein secretion – the focus of this Review.

**Box 3. Protein secretion in *D. discoideum***

In *D. discoideum*, two mechanistically different pathways, are known to facilitate protein secretion. The first, the so-called conventional pathway, transports proteins through the ER and Golgi complex, where they are packaged into vesicles and finally secreted (Viotti, 2016). Most proteins secreted via this pathway contain a secretion-signal peptide. The alternative unconventional pathway, which has been predicted to regulate the release of proteins that lack a secretion-signal peptide, involves the Golgi reassembly-stacking protein (GrpA) (Box 1) (Kinseth et al., 2007) and contractile vacuole (CV) system (Box 1) (Sesaki et al., 1997). However, some proteins that contain a secretion-signal peptide are secreted via the unconventional pathway (Nickel and Rabouille, 2009; Viotti, 2016).

Previous studies used liquid chromatography coupled with tandem mass spectrometry (LC–MS/MS) to show that ≥3% of proteins encoded by the *D. discoideum* genome are secreted, with the caveat that some of the detected proteins could have been from lysed cells or surface proteins that were released from the plasma membrane during sample collection (Bakthavatsalam and Gomer, 2010; Huber, 2017). Gene ontology (GO) term enrichment analysis (Box 1) showed that many of the secreted proteins are involved in metabolic and proteolytic processes. One route for secreted proteins is their deposition into the extracellular matrix (ECM). Following mound formation in *D. discoideum*, cells secrete material that eventually forms the slime sheath (i.e. the ECM) of the migrating slug (Huber and O'Day, 2017) (Fig. 1). LC–MS/MS has been used to identify >300 proteins in the slime sheath (Huber and O'Day, 2015). GO term analysis of the identified sheath proteins revealed an enrichment of proteins involved in binding, metabolism, catalysis and proteolysis. Together, these findings highlight our extensive knowledge of secreted proteins in *D. discoideum*. Combined with the genetic tractability of *D. discoideum*, they also demonstrate the ability to use *D. discoideum* to examine the effect of gene loss or mutation on targeted and global protein secretion.

## Secretion of NCL-related proteins in *D. discoideum* and mammals

Proteomics-based approaches revealed that *D. discoideum* homologs of five of the 11 human NCL-related proteins are secreted during *D. discoideum* development; these are the homologs of human palmitoyl protein thioesterase 1 (PPT1; *D. discoideum* Ppt1), tripeptidyl peptidase 1 (TPP1; *D. discoideum* Tpp1B, Tpp1C, Tpp1F), ceroid lipofuscinosis neuronal 5 (CLN5; *D. discoideum* Cln5), cathepsin D (CTSD; *D. discoideum* CtsD) and cathepsin F (CTSF; *D. discoideum* CprA, CprB, CprD, CprE, CprF, CprG, uncharacterized protein DDB0252831) (Bakthavatsalam and Gomer, 2010; Huber, 2017; Huber and Mathavarajah, 2018a,b) (Table 1). CLN5, CTSD and CTSF have also been detected extracellularly in mammals (Poole et al., 1973; Isosomppi et al., 2002; Hughes et al., 2014; Öörni et al., 2004; Kaakinen et al., 2007) and recent work identified PPT1 and TPP1 in human saliva (Kohan et al., 2005). In addition, previous work has confirmed that PPT1 (Lyly et al., 2007), TPP1 (Pal et al., 2009), and CLN5 (Moharir et al., 2013) and CTSD (Erickson et al., 1981) are glycosylated, which is a key attribute of secreted proteins. The glycosylation (Box 1) of CTSF has not been previously studied. Consistent with these findings, the bioinformatic tool SignalP 5.0 (Almagro Armenteros et al., 2019) detects putative secretion-signal peptides within human PPT1, TPP1, CLN5, CTSD and CTSF as well as the corresponding *D. discoideum* homologs. In mammals, the NCL-related protein progranulin (GRN) (Table 1) also contains a putative secretion-signal peptide, is glycosylated and has been detected extracellularly (Zhou et al., 1993). However, its *D. discoideum* homolog granulin (Grn), which also contains a putative secretion-signal peptide, has not been detected outside the cell (Bakthavatsalam and Gomer, 2010; Huber and O'Day, 2015; Huber, 2017), suggesting that extracellular Grn is unstable or binds to the cell surface shortly after being secreted.

In addition to their extracellular localization, all these NCL-related proteins also localize to lysosomes where they function as enzymes (Cárcel-Trullols et al., 2015; Huber and Mathavarajah, 2018a). These observations support previous work reporting the detection of lysosomal enzymes outside the cell and their emerging roles in disease development and progression (Vizovišek et al., 2019), suggesting that secreted NCL-related proteins function extracellularly. The detection of extracellular cathepsins (Box 1) in *D. discoideum* (Bakthavatsalam and Gomer, 2010; Huber, 2017), i.e. homologs of human CTSB, CTSD and CTSF, is also consistent with the importance of cathepsin activity in NCL pathology. For example, cathepsin activity is altered in leukocytes and cultured skin fibroblasts from CLN2 disease (Box 1) patients (Bennett et al., 1992), as well as in *CLN3*-depleted HeLa cells (Metcalf et al., 2008). Whereas the roles of secreted NCL-related proteins are not entirely clear, their detection outside cells indicates that they have important extracellular functions.

## Mechanisms that regulate Cln5 secretion in *D. discoideum*

**Fig. 2.**
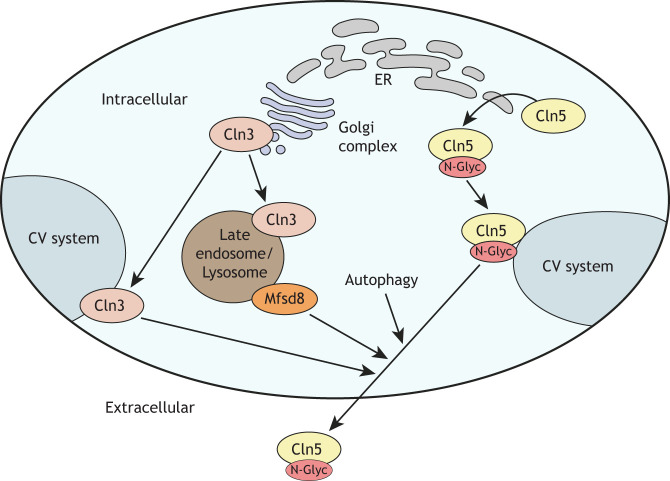
**Mechanisms regulating Cln5 secretion in *D. discoideum*.** Cln5 is glycosylated in the ER and trafficked to the CV system prior to secretion. Cln3 localizes to the Golgi complex and CV system. Cln3 also localizes to the late endosome/lysosome, as does Mfsd8. Secretion of Cln5 is regulated by Cln3 and Mfsd8 (i.e. loss of Cln3 or Mfsd8 increases Cln5 secretion), as well as autophagy (autophagy inhibition decreases Cln5 secretion). N-Glyc, N-glycosylation.

Mutations in *CLN5* primarily cause a late infantile-onset form of NCL known as CLN5 disease (Mole and Cotman, 2015). However, juvenile (Cannelli et al., 2007) and adult (Xin et al., 2010) cases have also been reported. In mammals, CLN5 is thought to localize primarily to the lysosome (Isosomppi et al., 2002; Moharir et al., 2013; Hughes et al., 2014). However, it has also been detected extracellularly in baby hamster kidney cells (Isosomppi et al., 2002) and mixed neural cells from *Cln5*-deficient sheep (Hughes et al., 2014). In *D. discoideum*, Cln5 is found in the endoplasmic reticulum (ER), at punctate distributions in the cytoplasm, in the contractile vacuole (CV) system, at the cell periphery and extracellularly (Huber and Mathavarajah, 2018a,b) (Fig. 2). Secretion of Cln5 in *D. discoideum* has been well studied, and has fostered new speculation into the localization and function of human CLN5. In *D. discoideum*, secretion of Cln5 is regulated by at least two other NCL-related proteins, Cln3 (Huber, 2017; Huber and Mathavarajah, 2018b) and major facilitator superfamily domain-containing protein 8 (Mfsd8), loss of which increases Cln5 secretion (Fig. 2) (Huber et al., 2020b). Mfsd8 is the *D. discoideum* homolog of human MFSD8 (Table 1) and mutations in *MFSD8* cause CLN7 disease (Box 1) (Table 1). In addition, recent work suggests that *D. discoideum* Cln5 is secreted following induction of autophagy via an unconventional pathway involving the CV system (Huber and Mathavarajah, 2018b; McLaren et al., 2021) (Fig. 2). Although the primary function of autophagy is to break down and recycle intracellular material, mounting evidence suggests it also participates in conventional and unconventional protein secretion (New and Thomas, 2019; Cavalli and Cenci, 2020; Padmanabhan and Manjithaya, 2020). While the mechanisms regulating autophagy-dependent secretion are not fully understood, autophagy (ATG) genes (Box 1), autophagosomes (Box 1) and multivesicular bodies (Box 1) are thought to play important roles (Duran et al., 2010; Manjithaya et al., 2010; Chen et al., 2017). Consistent with these findings, pharmacological treatment (ammonium chloride or chloroquine) or deletion of ATG genes (*atg1^−^* or *atg9^−^*) reduce Cln5 secretion in *D. discoideum* (Huber and Mathavarajah, 2018b; McLaren et al., 2021). In addition, the functions of *D. discoideum* Cln5 (McLaren et al., 2021), mouse CLN5 (Leinonen et al., 2017), sheep CLN5 (Best et al., 2017) and human CLN5 (Adams et al., 2019; Doccini et al., 2020) have all been linked to autophagy. Another important regulator of *D. discoideum* Cln5 secretion is glycosylation (Huber and Mathavarajah, 2018b); and, in human cells, CLN5 mislocalizes to the Golgi complex when glycosylation is inhibited (Moharir et al., 2013). Moreover, recent work revealed that *D. discoideum* Cln5 and human CLN5 have glycoside hydrolase activity (Huber and Mathavarajah, 2018a), and accumulated evidence suggests that CLN5 and the well-studied glycoside hydrolase β-hexosaminidase subunit alpha (HEXA) participate in the same biological pathway (Huber and Mathavarajah, 2018a; McLaren et al., 2021). In *D. discoideum*, the HEXA-like proteins N-acetylglucosaminidase A and B (NagA and NagB, respectively), are secreted during development (Bakthavatsalam and Gomer, 2010; Huber, 2017). Collectively, these observations indicate that additional work is necessary to resolve the primary localization of CLN5 in humans, and whether it functions in lysosomes, extracellularly or both.

## Role of Cln3 in conventional and unconventional protein secretion

NCL-related proteins are not only secreted themselves but can also regulate the secretion of other proteins. In *D. discoideum*, mutations in individual NCL-related genes result in aberrant protein secretion during growth and development. The best-studied example is Cln3, which is thought to regulate both conventional and unconventional protein secretion in *D. discoideum*. Human CLN3 contains multiple transmembrane domains but its precise function is not known (Mirza et al., 2019). In *D. discoideum*, *cln3* deficiency alters the extracellular amount of proteins that contain a secretion-signal peptide, as well as those that do not (Huber, 2017). Cln3 also localizes to both the Golgi complex (Huber, 2017) and CV system (Huber et al., 2014, 2017). The localization of Cln3 to the Golgi complex in *D. discoideum* is consistent with observations of CLN3 localization in yeast (Chattopadhyay et al., 2003; Codlin and Mole, 2009; Kama et al., 2011) and several mammalian cell lines (Kremmidiotis et al., 1999; Haskell et al., 1999; Kida et al., 1999; Persaud-Sawin et al., 2004; Metcalf et al., 2008; Tecedor et al., 2013). Collectively, these findings indicate that protein secretion is a conserved function of CLN3 across eukaryotes and that loss of this function in humans contributes to CLN3 disease (Box 1) pathology.

## *cln3* deficiency affects *D. discoideum* growth through protein secretion

**Fig. 3.**
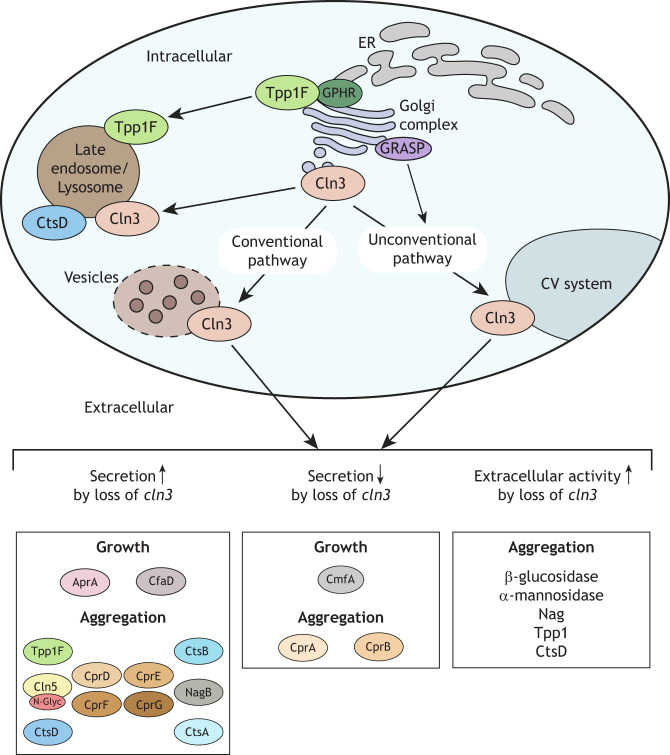
**Protein secretion regulated by Cln3 in *D. discoideum***. Cln3 localizes to the late endosome/lysosome, together with Tpp1F and CtsD. Tpp1F also localizes to the ER and Golgi complex, and binds GPHR. For secretion via the conventional pathway, proteins are transported via the ER and Golgi complex, where they are packaged into vesicles prior to secretion. The alternative unconventional pathway involves GRASP and the CV system. Cln3 localizes to both the Golgi complex and CV system to regulate protein secretion via these pathways. Loss of *cln3* increases the secretion of AprA and CfaD during growth, and Tpp1F, Cln5, CtsD, CprD, CprE, CprF, CprG, CtsB, NagB, and CadA during aggregation. Loss of *cln3* decreases the secretion of CmfA during growth, and CprA and CprB during aggregation. Loss of *cln3* increases the extracellular activity of beta-glucosidase, alpha-mannosidase, Nag, Tpp1, and CtsD during aggregation. Cpr, cysteine proteinase; GPHR, Golgi pH regulator (officially known as Gpr89); GRASP, Golgi reassembly-stacking protein (officially known as GrpA); N-Glyc, N-glycosylation; Tpp1F, tripeptidyl peptidase 1F.

During *D. discoideum* growth, *cln3* deficiency increases the rate of cell proliferation by affecting the secretion and cleavage of autocrine proliferation repressor A (AprA) (Huber et al., 2014) (Fig. 3). AprA negatively regulates cell proliferation (Brock and Gomer, 2005), shares structural and functional similarity with human dipeptidyl peptidase 4 (DPP4) (Box 1) (Herlihy et al., 2013, 2017), and is regulated by a pathway that involves homologs of proteins linked to NCL-related protein function in other model systems, e.g. extracellular signal-related kinases (ERKs), protein kinase A (PKA) and mechanistic target of rapamycin (MTOR) (Michalewski et al., 1998; Bakthavatsalam et al., 2009; Phillips and Gomer, 2010; Phillips et al., 2011; Bowman et al., 2011; Kanninen et al., 2013; Phillips and Gomer, 2014; Bond et al., 2015; Danyukova et al., 2018; Tang et al., 2018; Rijal et al., 2019). *cln3* deficiency also reduces the cleavage of extracellular AprA (Huber et al., 2014) (Fig. 3), suggesting that loss of *cln3* decreases the secretion of a protease that processes AprA. This was confirmed by a follow-up study that reported altered secretion of several cysteine proteases by *cln3^−^* cells (Huber, 2017). *cln3* deficiency also increases the intracellular amount of counting factor-associated protein D (CfaD) during the early log phase of axenic growth (Huber et al., 2014) (Fig. 3). CfaD is like cathepsin L (CTSL) from various species and part of an extracellular complex that binds AprA (Bakthavatsalam et al., 2008). This suggests that, like AprA, altered secretion of CfaD contributes to the increased proliferation of *cln3^−^* cells. Together, these results suggest a link between the enhanced proliferation of *cln3^−^* cells and aberrant secretion of extracellular signaling proteins. Intriguingly, recent work has also revealed how loss of *tpp1* (Smith et al., 2019) or *cln5* (McLaren et al., 2021) affects cell proliferation, suggesting that altered protein secretion also underlies growth stage phenotypes in these knockout cell lines. However, in *tpp1^−^* and *cln5^−^* cell lines, proliferation is reduced not increased, suggesting that NCL-related proteins serve different regulatory functions during *D. discoideum* growth.

## *cln3* deficiency affects adhesion and aggregation of *D. discoideum* through protein secretion

Aberrant protein secretion has also been linked to the delayed aggregation of *D. discoideum cln3^−^* cells. During growth, loss of *cln3* reduces the extracellular amount of conditioned medium factor A (CmfA) (Huber, 2017) (Fig. 3), a glycoprotein that enables starving cells to respond to pulses of cAMP (Yuen et al., 1991). These findings, coupled with the effect of *cln3* deficiency on AprA secretion and cleavage during growth, suggest that the delayed aggregation is due to *cln3^−^* cells not being primed to enter the developmental program owing to alterations in cell–cell signaling. *cln3* deficiency also reduces cell–cell and cell–substrate adhesion (Huber et al., 2017), both of which are essential for aggregation during the early stages of development. The *D. discoideum* genome encodes the well-characterized calcium-dependent cell-adhesion protein A (CadA), which shares limited sequence similarity with classic cadherins (Knecht et al., 1987; Wong et al., 1996; Brar and Siu, 1993). During development, CadA is secreted by the CV system (Sesaki et al., 1997; Bakthavatsalam and Gomer, 2010; Sriskanthadevan et al., 2013; Huber, 2017) and present in the slug ECM (Huber and O'Day, 2015). High amounts of extracellular CadA have anti-adhesive effects (Siu et al., 1997) and, during aggregation, *cln3* deficiency increases the extracellular amount of CadA (Huber et al., 2017) (Fig. 3), suggesting that loss of *cln3* reduces the amount of membrane-tethered CadA, thus compromising adhesion. Deficiency of *cln3* also reduces cAMP-mediated chemotaxis (Huber et al., 2017). When starved, *D. discoideum* cells produce and secrete cAMP, which acts as a chemoattractant for multicellular aggregation (Mathavarajah et al., 2017). Although loss of *cln3* has no effect on the expression or localization of cAMP signal transduction proteins (Huber, 2017), it does affect the expression and intracellular amount of AprA (Huber and Mathavarajah, 2019), which functions as a chemorepellent during development (Phillips and Gomer, 2012). Therefore, in addition to cell proliferation, the reduced chemotaxis of *cln3^−^* cells might also be linked to altered levels of AprA. Intriguingly, recent work reported chemotaxis, adhesion and aggregation defects for *cln5^−^* cells (Huber and Mathavarajah, 2018b; McLaren et al., 2021), along with an altered extracellular amount of CadA (McLaren et al., 2021). Together, these findings provide additional evidence that link NCL-related gene loss to aberrant protein secretion during *D. discoideum* development and suggest that protein secretion is, indeed, altered in multiple NCL subtypes.

## Comparative transcriptomics links Cln3 to protein secretion during aggregation of *D. discoideum*

RNA sequencing was used to determine the effects of *cln3* deficiency on gene expression during aggregation of *D. discoideum* (Huber and Mathavarajah, 2019). GO term analysis identified enrichment of differentially expressed genes encoding proteins that localize to the cell periphery, plasma membrane and extracellular space. In Cln3-deficient cells, RNA sequencing also revealed reduced expression of genes encoding Rab11B, syntaxin 1B, vacuolin B and vacuole membrane protein 1 (Vmp1), all of which are associated with secretion (Huber and Mathavarajah, 2019). Interestingly, Vmp1 shares many attributes with Cln3, including localization to the Golgi complex and CV system, and functioning in cell proliferation, aggregation and osmoregulation (Calvo-Garrido et al., 2008, 2014; Calvo-Garrido and Escalante, 2010). GO term enrichment analysis also revealed enrichment of differentially expressed genes linked to catalytic activity (Huber and Mathavarajah, 2019). For example, *cln3* deficiency decreases the expression of genes encoding the lysosomal enzymes beta-glucosidase, alpha-mannosidase and N-acetylglucosaminidase (Huber and Mathavarajah, 2019). Loss of *cln3* also reduces the intracellular activity of beta-glucosidase and alpha-mannosidase, and increases the extracellular activity of those enzymes as well as N-acetylglucosaminidase (Huber and Mathavarajah, 2019) (Fig. 3), suggesting that *cln3^−^* cells increase secretion of these lysosomal enzymes. Based on these observations, it might be that cells reduce the expression of genes that encode beta-glucosidase, alpha-mannosidase and N-acetylglucosaminidase, to counteract the increased secretion and extracellular activity of these enzymes.

The trafficking and secretion of lysosomal enzymes has been well studied in *D. discoideum*. During growth and aggregation, *D. discoideum* secretes precursor and mature forms of lysosomal enzymes including beta-glucosidase, alpha-mannosidase, and N-acetylglucosaminidase (Dimond et al., 1981; Mierendorf et al., 1985; Cardelli et al., 1990). The release of lysosomal enzymes is due to an active secretory process, not passive leakage of enzymes from cells or their incidental release during the egestion of digested material (Dimond et al., 1981). Previous work suggests that sorting of secreted and intracellular pools of lysosomal enzymes occurs in the Golgi complex (Mierendorf et al., 1985), and that protease activity is required for trafficking enzymes to lysosomes (Richardson et al., 1988). Intriguingly, Cln3 localizes to the Golgi complex in *D. discoideum* and influences the secretion of several lysosomal enzymes (Huber, 2017). These findings suggest that loss of *cln3* affects the cleavage of precursor forms of lysosomal enzymes, and/or the sorting of secreted and intracellular pools of lysosomal enzymes at the Golgi complex. Thus, work on Cln3 has provided new insight on the mechanisms that regulate lysosomal enzyme secretion. Overall, comparative transcriptomics has provided further support for a role of Cln3 in protein secretion, and suggests that altered secretion plays an important role in the development and progression of CLN3 disease.

## Mass spectrometry links Cln3 to protein secretion during *D. discoideum* aggregation

An LC–MS/MS analysis of *cln3^−^* conditioned buffer (Box 1) solidified the role of Cln3 in protein secretion during aggregation (Huber, 2017). GO term analysis revealed an extracellular enrichment of proteins associated with vesicle-mediated transport, endocytosis, proteolysis and metabolism. Among the affected proteins are the *D. discoideum* homologs of human TPP1 (Tpp1F, increased), CLN5 (Cln5, increased), CTSD (CtsD, increased), CTSF (CprA, decreased; CprB, decreased; CprD, increased; CprE, increased; CprF, increased; CprG, increased), CTSB (CtsB, increased), and HEXA (NagB, increased) (Huber, 2017). Loss of *cln3* also reduces the expression and intracellular activity of CtsD and increases CtsD activity in conditioned buffer (Huber and Mathavarajah, 2019) (Fig. 3), suggesting increased secretion of this cathepsin. These findings suggest that *cln3^−^* cells compensate for the increased secretion and extracellular activity of CtsD by reducing *ctsD* expression. In addition, *cln3* deficiency increases TPP1 activity in conditioned buffer (Huber and Mathavarajah, 2019), which aligns with an increased amount of Tpp1F outside *cln3^−^* cells (Huber, 2017) (Fig. 3). These findings are consistent with the previously described effects of *cln3* deficiency on the expression of genes encoding lysosomal enzymes (Huber and Mathavarajah, 2019). Previous work in *D. discoideum* showed that enzymes secreted during the early stages of development facilitate adhesion and aggregation (Rossomando et al., 1978; Dimond et al., 1981; Ebert et al., 1990). Therefore, the combined effect of *cln3* deficiency on the expression and secretion of lysosomal enzymes might contribute to the reduced adhesion and delayed aggregation of *cln3*-deficient cells (Huber et al., 2017).

Accumulated evidence suggests that *cln3* deficiency deregulates protein secretion, since several proteins that are not normally secreted by wild-type (WT) cells can be detected in *cln3^−^* conditioned buffer (Bakthavatsalam and Gomer, 2010; Huber and O'Day, 2015; Huber, 2017) and most aberrantly secreted proteins are present at increased, not decreased, amounts in conditioned buffer (Huber, 2017). In support of this hypothesis, our group reported an enrichment of downregulated genes in *cln3^−^* cells, whose protein products localize extracellularly (Huber and Mathavarajah, 2019). This observation could reflect the cell downregulating the expression of genes whose protein products are present extracellularly at abnormally high levels.

## Altered protein secretion in *D. discoideum* NCL-related gene knockout models

Recent work in *D. discoideum* suggests that multiple NCL-related proteins may function in protein secretion. As discussed previously, Cln5 and CtsD are glycosylated and secreted during *D. discoideum* development (Huber and Mathavarajah, 2018a,b; Journet et al., 1999), which aligns with observations of secreted CLN5 and CTSD in mammals (Isosomppi et al., 2002; Hughes et al., 2014; Poole et al., 1973) (Table 1). Like *cln3^−^* cells, loss of *mfsd8* in *D. discoideum* also increases the secretion of Cln5 (Fig. 2) and CtsD (Huber et al., 2020b). The effects of *mfsd8* deficiency on protein secretion in *D. discoideum* are consistent with the role of MFSD8 in lysosome exocytosis observed in an immortalized cell line generated from cerebellar granule neuron precursors (Box 1) isolated from *Cln7^−/−^* mice (von Kleist et al., 2019). In addition, like *cln3^−^* cells, loss of *cln5* in *D. discoideum* also affects the amount of CadA outside the cell, which is thought to play a role in the chemotaxis, adhesion and aggregation defects observed for *cln5^−^* cells (McLaren et al., 2021). In mammals, recent findings associate DNAJC5 – which causes CLN4 disease (Box 1) when mutated (Mole and Cotman, 2015) – with misfolding-associated protein secretion (Box 1) (Xu et al., 2018). In addition, loss of *TPP1* in a canine model of CLN2 disease damages cells, causing the release of cardiac troponin-1, alanine aminotransferase and creatine kinase into blood plasma (Katz et al., 2017). Combined, these findings suggest that multiple NCL-related proteins regulate protein secretion, which, ultimately, might play an important role in the development and progression of the NCLs.

## Potential biomarkers identified in animal models and humans

Most NCL patients receive their diagnosis only after the first symptoms are detected. For childhood diseases, such as the NCLs, newborn or infantile diagnosis may present an opportunity to administer therapies that prevent or slow down disease progression. The classic diagnosis for NCL involves microscopic analysis of a skin biopsy for accumulation of ceroid lipofuscin (Williams et al., 2006). Genetic testing and enzyme activity assays are also available for NCL subtypes caused by loss of a specific lysosomal enzyme (e.g. TPP1 activity in CLN2 disease). Unfortunately, existing tests cannot monitor disease progression or the patients' responses to treatment. Based on these limitations, studies in animal models and humans have begun to explore the protein content of cerebrospinal fluid (CSF) and urine to identify potential diagnostic, prognostic and therapeutic-response biomarkers, which could also help to optimize dosage for patients undergoing treatment. Some of the findings from these studies align with observations in *D. discoideum*, strongly supporting the role of aberrant protein secretion in NCL pathology. The sections below describe recent studies of altered protein secretion in animal models, followed by a discussion of similar work using human samples. Collectively, these studies have laid the foundation for future work to identify and validate potential NCL biomarkers.

### Altered protein secretion in neural cells derived from a CLN3 disease mouse model

*Cln3*-deficient murine microglia (Box 1) and astrocytes (Box 1) have altered protein secretion profiles (Parviainen et al., 2017). In microglia, altered secretion is observed only after stimulation with lipopolysaccharide, not under basal conditions. Three chemokines (i.e. CCL5, CCL9, CXCL2) (Box 1), matrix metalloproteinase 9 (MMP9) and the glycoprotein von Willebrand factor (VWF) are all secreted at significantly reduced levels. Under basal conditions, *Cln3*-deficient astrocytes secrete reduced amounts of the chemokines CCL9 and granulocyte chemotactic protein 2 (GCP-2, CXCL5), and an increased amount of tissue factor (Box 1). However, when activated by lipopolysaccharide and interferon gamma, secretion of several proteins by *Cln3*-deficient astrocytes is significantly reduced, including that of mitogens, chemokines, anti- and pro-inflammatory cytokines and glutathione (Box 1) compared to secretion by WT cells (Ross et al., 2012). This altered secretion points to an important role of CLN3 in regulating the immune function of astrocytes, as its loss impairs the release of immunomodulators. Many of the proteins detected at reduced amounts also have neuroprotective properties (Azizi et al., 2014; Wang et al., 2015). Since neuroinflammation is a common hallmark of the NCLs and can be used to predict neuron loss (Behnke and Langmann, 2021), the altered secretion of neuroprotective proteins might compromise the health and viability of neurons in brains of patients diagnosed with CLN3 disease. By contrast, *Cln3*-deficient astrocytes more readily secreted fibrinogen and the mitogen C-reactive protein (CPR). Since fibrinogen and CPR are also neuroprotective (Jeon et al., 2021), their increased extracellular presence could reflect an attempt by *Cln3*-deficient astrocytes to counteract the neurodegenerative process. Together, these data indicate that cell–cell communication through secreted factors is perturbed in brain from a CLN3 diesease mouse model, which is consistent with observations of altered signaling in *D. discoideum cln3^−^* cells (Huber et al., 2014, 2017; Huber, 2017).

### Altered protein secretion in neural cells derived from a CLN6 disease mouse model

As discussed above, previous work in *D. discoideum* revealed proteins whose secretion is affected by *cln3* deficiency (Huber, 2017). Using a similar approach, recent work examined the secretome of a CLN6 disease mouse model (*Cln6^nclf^*), by co-culturing primary cortical cell cultures of *Cln6^nclf^* neurons, microglia and astrocytes, followed by an analysis of the protein content of conditioned medium (Box 1) (Best et al., 2021). Conditioned medium from WT cells contained 152 proteins, whereas conditioned medium from *Cln6^nclf^* cells contained 169 proteins. Of the latter, 47% contained a putative secretion-signal peptide and 22% were secreted via non-classic means. The remaining 31% of proteins were predicted to be secreted by unconventional mechanisms or in response to some form of cellular stress (e.g. inflammation) (Best et al., 2021). These results are consistent with the presence of proteins in conditioned buffer of *D. discoideum cln3^−^* cells, which are normally not secreted and do not have a putative secretion-signal peptide (Huber, 2017). Best and colleagues found 37 proteins whose levels are significantly increased in *Cln6^nclf^* conditioned medium, which might reflect a compensatory or protective response by *Cln6^nclf^* cells. STRING (Box 1) and GO term enrichment analyses of proteins detected in *Cln6^nclf^* conditioned medium align with previous observations in *D. discoideum cln3^−^* cells. For example, STRING analysis highlighted a cluster of proteins associated with proteolysis. More specifically, *Cln6^nclf^* conditioned medium contained increased levels of the proteases CTSB, CTSD and CTSL (Best et al., 2021), which is consistent with earlier findings in *D. discoideum cln3^−^* (Huber, 2017) (Fig. 3) and *mfsd8^−^* cells (Huber et al., 2020b), as well as in mammalian models of CLN2 and CLN3 disease (Bennett et al., 1992; Metcalf et al., 2008). STRING analysis also revealed a cluster of proteins associated with development of the nervous system. Whereas the mechanisms regulating multicellular development in *D. discoideum* and nervous system development in mammals are quite different, the cluster of proteins associated with nervous system development secreted in *Cln6^nclf^* murine cell culture conditioned medium (Best et al., 2021) mirrors work in *D. discoideum* linking the functions of *tpp1A* (Phillips and Gomer, 2015), *cln3* (Huber et al., 2014) and *cln5* (McLaren et al., 2021) to developmental timing. These results are also in agreement with studies that have linked NCL-related proteins to a variety of developmental processes in mammals (Herrmann et al., 2008; Fabritius et al., 2014; Savchenko et al., 2017; Singh et al., 2019; Connolly et al., 2019).

GO term analysis of proteins detected in *Cln6^nclf^* conditioned medium revealed an enrichment of proteins associated with catalytic activity, metabolism, transport and cell adhesion. Like *Cln6^nclf^* cells, *cln3* deficiency in *D. discoideum* also affects the secretion of proteins involved in metabolism and transport (Huber, 2017). In addition, the increased secretion of transport proteins by *Cln6^nclf^* cells is consistent with the role of WT CLN6 in the transport of lysosomal enzymes (Bajaj et al., 2020). The identification of protein clusters in *Cln6^nclf^* conditioned medium associated with cell adhesion also aligns with the compromised adhesion of *D. discoideum cln3^−^* (Huber et al., 2017) and *cln5^−^* (Huber and Mathavarajah, 2018b) cells, suggesting that altered adhesion underlies multiple forms of NCL. β-hexosaminidase subunit beta (HEXB) is also among the 37 proteins present in increased amounts in *Cln6^nclf^* conditioned medium (Best et al., 2021). Similarly, NagB, a HEXB-like protein, is also increased in *D. discoideum cln3^−^* conditioned buffer (Huber, 2017) (Fig. 3).

Restoring *Cln6* expression by using viral-mediated gene therapy partially corrected the increased extracellular amounts of angiotensin (Box 1), high endothelial venule protein (Box 1), 14-3-3 protein zeta/delta (Box 1), cationic trypsinogen (Box 1) and the chaperone heat shock protein 90 (Box 1) in *Cln6^nclf^* conditioned medium, as well as the increased extracellular activity of CTSL (Best et al., 2021) supporting the use of these proteins as biomarkers in short-term therapeutic studies. Overall, Best et al. (2021) identified a set of potential biomarkers for CLN6 disease that can be evaluated further.

### CSF from CLN1, CLN2 and CLN3 disease mouse models

Three NCL mouse models, i.e. *Ppt1^−/−^*, *Tpp1^−/−^* and *Cln3*^−/−^ (Table 1), were used to examine the potential of CSF to provide viable biomarkers (Sleat et al., 2019). Samples were collected and analyzed at early- and late-stage of the disease. Few changes were observed at early pre-symptomatic timepoints. However, later in the disease course, multiple proteins were altered in CSF obtained from CLN1 disease (Box 1) and CLN2 disease mice, suggesting that the changes are the consequence of progressive neurodegeneration rather than an immediate biological response due to mutations in *Ppt1* or *Tpp1*, respectively. Very few alterations were observed in CSF collected from early- or late-stage CLN3 disease mice, which could be explained by these mice presenting no overt end-stage phenotype and having a normal lifespan (Sleat et al., 2019). In general, a wider range of proteomic changes was observed in CSF from CLN1 disease mice compared to that from CLN2 disease mice, suggesting that pathology is more widely distributed in the brains of CLN1 disease mice. However, the comparatively smaller range of changes in CLN2 disease mice might be a consequence of localized, highly damaging pathology in only certain regions of the brain, which is consistent with the shorter lifespan of CLN2 disease mice (Sleat et al., 2019).

Many of the altered proteins in CSF from CLN1 and CLN2 disease mice are lysosomal in origin or markers of neuroinflammation (Sleat et al., 2019). In addition, all lysosomal proteins found to be altered in CSF were detected at increased amounts. In CSF from late-stage CLN1 disease mice, levels of apolipoprotein E (APOE), CTSD, CTSZ, HEXA, and HEXB are significantly elevated. HEXA and HEXB levels are elevated at the early stage of CLN1 disease. In CSF from CLN2 disease mice, levels of APOE, CTSD and HEXB are significantly increased during early and late stages of the disease, whereas CTSZ is increased during late-stage disease only. CSF collected from CLN2 disease mice also contains increased amounts of neurofilament heavy polypeptide and neurofilament medium polypeptide. These findings are intriguing as these proteins were also detected in CSF and blood plasma from Alzheimer's and Parkinson's disease patients (Khalil et al., 2018; Lin et al., 2019; Didonna and Opal, 2019; Preische et al., 2019). Although neurofilaments are intermediate filaments that primarily provide structural support for neurons (Yuan et al., 2017), recent work also highlights their presence in synapses and their potential role in neurotransmission (Yuan et al., 2015). Through their work, Sleat and colleagues highlighted the potential of CSF to provide biomarkers for the NCLs and identified several proteins that should be further evaluated in future studies (Sleat et al., 2019).

### Blood plasma from NCL patients

LC–MS/MS performed on blood plasma from CLN1, CLN2, CLN3 and CLN5 disease patients identified adiponectin, APOE, brain-derived neurotrophic factor (BDNF), neuronal cell adhesion molecule (NRCAM), vascular cell adhesion molecule 1 (VCAM1), clusterin and myoglobin as potential biomarkers (Hersrud et al., 2016). Adiponectin and APOE are involved in lipid metabolism (Danielsson et al., 1978; Nakano et al., 1996). Adiponectin also plays an anti-inflammatory role in the cardiovascular system (Zoccali et al., 2004), which is interesting given that cardiovascular defects are frequently reported in CLN3 disease patients (Rietdorf et al., 2020). In addition, increased levels of adiponectin have been reported in CSF from patients diagnosed with multiple sclerosis (Hietaharju et al., 2010) or Alzheimer's disease (Une et al., 2011), and APOE is associated with a genetic predisposition to late-onset Alzheimer's disease (Burke and Roses, 1991-1992). However, APOE also serves many important functions in healthy brain (Flowers and Rebeck, 2020). Whereas *APOE* is expressed most in the adrenal gland and liver (Uhlén et al., 2015; Human Protein Atlas), astrocytes and microglia produce and secrete APOE into CSF to facilitate cholesterol transport to neurons (de Chaves and Narayanaswami, 2008). Therefore, alterations in its extracellular levels could reflect a damaged blood–brain barrier. Intriguingly, as discussed earlier, APOE is aberrantly secreted in mouse models of CLN1 (Sleat et al., 2019), CLN2 (Sleat et al., 2019) and CLN6 disease (Best et al., 2021).

The neurotrophin BDNF has a neuroprotective role and is upregulated in response to neuroinflammation and brain injury (Lima Giacobbo et al., 2019). In addition, it can cross the blood–brain barrier during disease (Pan et al., 1998) and has been explored as a potential biomarker for several neurological diseases (Colucci-D'Amato et al., 2020). In NCL, the increased amount of BDNF in blood plasma could be due to the inflammatory response or a compensatory mechanism to counteract brain injury. Intriguingly, like adiponectin, BDNF also plays an important role in the cardiovascular system, which is impacted in some patients diagnosed with CLN3 disease (Hang et al., 2021; Rietdorf et al., 2020).

Clusterin is an extracellular protein chaperone that is activated during inflammation and disease (Satapathy and Wilson, 2021). However, its relevance to NCL is unclear since it is one of the most broadly identified biomarkers in a variety of diseases (Rodríguez-Rivera et al., 2021). Another potential biomarker, myoglobin, is released into the circulation upon muscle damage, including from the myocardium (Guerrero-Hue et al., 2018), which is consistent with the cardiac defects associated with CLN3 disease (Rietdorf et al., 2020). A recent study reported increased levels of myoglobin in the blood serum of patients with spinal and bulbar muscular atrophy but not in individuals with amyotrophic lateral sclerosis (Guo et al., 2021), suggesting that myoglobin is an appropriate biomarker for other neurological diseases. NRCAM and VCAM1 are members of the immunoglobin gene superfamily of adhesion proteins that regulate cell adhesion in the brain (Sakurai, 2012). In support of their altered levels in blood plasma, *D. discoideum cln3^−^* cells (Huber et al., 2017; Huber, 2017) and neural cells derived from a mouse model of CLN6 disease (Best et al., 2021) also aberrantly secrete adhesion proteins, as discussed earlier. In addition, loss of *cln3* (Huber et al., 2017) or *cln5* (Huber and Mathavarajah, 2018b) in *D. discoideum* causes adhesion defects. Increased NRCAM levels have been reported in the blood plasma of patients diagnosed major depressive disorder (Liu et al., 2021), and the function of NRCAM has been associated with Alzheimer's disease (Hu et al., 2010). However, recent work suggests that NRCAM is not a suitable CSF biomarker for differentiating between different forms of dementia (Müller et al., 2015); therefore, its use as an NCL biomarker requires further evaluation. Increased levels of VCAM1 have been detected in the blood plasma of a CLN1 disease mouse model, as well as in several non-NCL mouse models of lysosomal storage diseases (Woloszynek et al., 2007). VCAM1 has also been linked to multiple autoimmune (da Rosa Franchi Santos et al., 2020) and neurodegenerative diseases, such as Alzheimer's (Trombetta et al., 2018) and Parkinson's disease (Yu et al., 2020), pointing to potential relevance in the NCLs. In total, Hersrud and colleagues identified seven potential biomarkers for the NCLs in human blood plasma that warrant further examination (Hersrud et al., 2016).

### CSF from CLN1, CLN2 and CLN3 disease patients

Recent work used LC–MS/MS to analyze the protein content of autopsy brain and matching CSF from CLN1, CLN2 and CLN3 disease patients (Sleat et al., 2017). Four proteins were identified across all three disease subtypes; they were – at increased levels – aldehyde dehydrogenase 1 family member A2 (ALDH1A2), collagen type XIV alpha 1 chain (COL14A1) and cellular retinoic acid binding protein 1 (CRABP1), and – at a decreased level – carnosine dipeptidase 1 (CNDP1). However, other affected proteins appeared to be specific for certain subtypes of NCL. Calcium signaling is thought to play an important role in NCL pathology (Mathavarajah et al., 2018b) and the calcium-binding protein calbindin 1 (CALB1) was significantly elevated in CSF from CLN2 and CLN3 disease patients (Sleat et al., 2017). Increased amounts of CALB1 have also been reported in CSF obtained from patients with Niemann–Pick type C1 disease, another lysosomal storage disease (Bradbury et al., 2016). Consistent with these results, higher amounts of calcium-dependent cell adhesion protein CadA were detected in conditioned buffer from *D. discoideum cln3^−^* cells (Huber et al., 2017) (Fig. 3).

Subunit C of mitochondrial ATP synthase (SCMAS), which has been previously detected in urine from NCL patients (Wisniewski et al., 1994, 1995), was present in four out of the five CSF samples from CLN2 disease patients (Sleat et al., 2017), suggesting that it is a biomarker for CLN2 disease. Intriguingly, SCMAS is a component of the lysosomal storage material observed in NCL patients (Palmer, 2015). In total, Sleat and colleagues provided a useful starting set of candidate biomarkers and laid the groundwork for examining CSF as a source of viable NCL biomarkers (Sleat et al., 2017).

### Urine from NCL patients and NCL sheep models

Urine is attractive for biomarker identification because collection is inexpensive, non-invasive and can be obtained at frequent intervals in large volumes. NCL-related genes are expressed in all tissues (Uhlén et al., 2015; Human Protein Atlas); however, some NCL-related genes, such as *CLN3* (Stein et al., 2010), *CLN5* (Savukoski et al., 1998) and *MFSD8* (Damme et al., 2014), display particularly high expression levels in the kidney. In addition, accumulated evidence from a variety of cells (e.g. *D. discoideum*, baby hamster kidney cells, mouse kidney epithelial cells, mouse brain endothelial cells) suggests an important role of CLN3 in osmoregulation (Mathavarajah et al., 2018a; Getty et al., 2013; Stein et al., 2010; Tecedor et al., 2013). With that in mind, recent work used mass spectrometry to examine urine collected from CLN5 and CLN6 disease sheep models, as well as from NCL patients (Iwan et al., 2020). In urine collected from patients diagnosed with CLN2 disease, activation of the Bcl-2-associated athanogene 2 (BAG2) (Box 1), neuroinflammation and synaptogenesis pathways was increased; by contrast, the natural killer cell signaling pathway was inhibited. GO term analysis showed enrichment of proteins derived from the lysosome. The changes observed in urine from CLN5 and CLN6 disease sheep models were more subtle. In urine obtained from CLN5 disease sheep, effects on pathways related to carbohydrate metabolism were observed, which aligns with the glycoside hydrolase activity of CLN5 (Huber and Mathavarajah, 2018a; McLaren et al., 2021). In urine obtained from CLN6 disease sheep, the ER stress response, unfolded protein response and immune response pathways were affected (Iwan et al., 2020). Finally, like conditioned medium collected from neural cells derived from CLN6 disease mice (Best et al., 2021), urine collected from CLN6 disease sheep also contained abnormal amounts of calreticulin (CALR) and CTSB (Iwan et al., 2020). More specifically, CTSB levels were increased in both mouse and sheep, whereas CALR was increased in mouse and decreased in sheep.

Following the discovery phase discussed above, Iwan and colleagues analyzed urine collected from CLN1, CLN2, CLN3, CLN5, CLN6, and CLN7 disease patients (Iwan et al., 2020). Unfortunately, they found no correlation between proteins affected in sheep CLN5 or CLN6 disease models and humans diagnosed with CLN5 or CLN6 disease. However, several proteins were altered among the NCL subtypes, including HEXA (increased in patients diagnosed with CLN1, CLN3 and CLN5 disease, and in some patients diagnosed with CLN2 and CLN6 disease), LAMP1 (increased in patients diagnosed with CLN3 and CLN5 disease, and in some patients diagnosed with CLN2 disease), GOT1 (increased in patients diagnosed with CLN3 and CLN5 disease, and in some patients diagnosed with CLN2 disease), and TPP1 (reduced in all NCL subtypes). In summary, this work highlights the potential for urine to provide biomarkers for the NCLs and identified several candidates that warrant further investigation.

### Evaluation of potential biomarkers for the NCLs

The findings from studies summarized above were collated to reveal potential biomarkers for further evaluation (see Table S1 for full list). For example, an increased amount of APOE was detected in CSF from CLN1 and CLN2 disease mice (Sleat et al., 2019), in conditioned medium from cultured cells derived from CLN6 disease mice (Best et al., 2021), and in blood plasma collected from CLN1, CLN2, CLN3 and CLN5 disease patients (Hersrud et al., 2016). Increased levels of CTSZ were detected in CSF collected from CLN1 and CLN2 disease mice (Sleat et al., 2019), as well as in urine collected from CLN2 disease patients (Iwan et al., 2020). HEXA was increased in CSF from CLN1 disease mice (Sleat et al., 2019), and in urine collected from CLN1, CLN2, CLN3, CLN5 and CLN6 disease patients (Iwan et al., 2020). In addition, as discussed above, abnormal amounts of CALR and CTSB were detected in conditioned medium collected from cultured cells derived from CLN6 disease mice (Best et al., 2021) and in urine from CLN6 disease sheep (Iwan et al., 2020). Collectively, these findings suggest that CALR and CTSB can be used as effective biomarkers for CLN6 disease, whereas APOE, CTSZ and HEXA might serve as effective biomarkers for multiple NCL subtypes. Finally, abnormal amounts of ALDH1A2 (increased), COL14A1 (increased), CRABP1 (increased) and CNDP1 (decreased) are present in CSF from CLN1, CLN2, and CLN3 disease patients (Sleat et al., 2017), suggesting that these proteins can also be used as effective biomarkers.

Although this Review highlights several findings that have been observed across multiple models (i.e. *D. discoideum*, mouse and sheep) as well as in samples obtained from NCL patients, it is important to note that not all putative biomarkers identified in model systems will translate to humans. For example, some proteins that are aberrantly secreted in different mouse models of NCL (Sleat et al., 2019) have not yet been shown to be aberrantly secreted in humans (Sleat et al., 2017). The levels of several proteins, including complement C4-B (C4B), CTSD, HEXB, lysozyme C2 (LYZ2) and serine protease inhibitor A3N (SERPINA3N) are increased in CSF from CLN1 and CLN2 disease mice (Sleat et al., 2019), as well as in conditioned medium collected from cultured neural cells derived from CLN6 disease mice (Best et al., 2021). The *D. discoideum* homolog of human CTSD, CtsD, is also aberrantly secreted by *D. discoideum cln3^−^* (Huber, 2017) (Fig. 3) and *mfsd8^−^* cells (Huber et al., 2020b). However, these alterations were not observed in the studies of human samples described in this Review (Hersrud et al., 2016; Sleat et al., 2017; Iwan et al., 2020). This does not infer that secretion of these proteins is not altered in NCL patients but simply that additional work is required to examine their relevance to humans.

Another observation is the lack of correlation between NCL subtypes in the secreted protein profile of the same cell type from the same organism. For example, no shared proteins were detected in conditioned medium from astrocytes and microglia obtained from mouse models of CLN3 (Parviainen et al., 2017) and CLN6 disease (Best et al., 2021). This suggests that each subtype of NCL requires its own unique set of biomarkers in a clinical setting. In addition, a comparison of proteins in CSF from mouse models of CLN1, CLN2 and CLN3 disease, and in CSF from patients with the same NCL subtypes, revealed that only CSF from CLN1 disease mice (Sleat et al., 2019) and human CLN1 disease patients (Sleat et al., 2017) contained shared proteins. However, this observation might not be reflective of all NCL subtypes. Since only CLN1, CLN2 and CLN3 disease samples were examined, shared proteins across other NCL subtypes might be observed in future studies of CSF obtained from other NCL models. Nonetheless, the detection of shared proteins in CSF from CLN1 disease mice and patients suggests they are valuable biomarkers. Finally, in mouse models of CLN1 and CLN2 disease, more alterations in the protein content of CSF were observed later in the disease course compared to pre-symptomatic timepoints (Sleat et al., 2019). This indicates that proteins identified during late-stage disease are not optimal biomarkers and, therefore, the focus should be on proteins that are affected earlier in the disease course.

## Potential role of altered protein secretion in the onset and progression of the NCLs

NCL diagnosis can range from congenital (e.g. CLN10 disease, mutations in *CTSD*) to juvenile (e.g. CLN3 disease, mutations in *CLN3*) and adult (e.g. CLN4 disease, mutations in *DNAJC5*). Whereas many NCL-related proteins function as lysosomal enzymes, others are proposed to regulate intracellular trafficking and transport across membranes (Cárcel-Trullols et al., 2015), which could explain the range of disease onset observed across the 13 subtypes of NCL. Mutations in essential lysosomal enzymes, i.e. CTSD, are likely to cause ceroid lipofuscin to accumulate at an increased rate and, thus, accelerate disease onset. However, other NCL-related proteins that participate in alternative pathological mechanisms – as for example, CLN3, which is not a lysosomal enzyme but, rather, plays a role in protein secretion – or whose cellular function can be partially restored by other proteins, would be associated with a more delayed disease onset.

An important question to consider is the precise role of altered protein secretion in NCL pathology. Is it a secondary effect of NCL-causing mutations or does it cause the accumulation of ceroid lipofuscin directly? If the former, studying altered protein secretion in the NCLs has clinical value for the development of biomarkers for NCL diagnosis, prognosis and therapy evaluation. However, if the latter, further studies of how aberrant protein secretion impacts NCL pathology is warranted to better understand how secretory pathways could be targeted therapeutically. For example, increased enzyme secretion in the NCLs might deplete lysosomes of essential enzymes required to degrade internalized material, resulting in the accumulation of this material within lysosomes. It might also disrupt intracellular signaling by affecting the structure and/or composition of the ECM (Parker and Kohler, 2010). Additionally, mutations in NCL-related genes might affect the secretion of extracellular proteins that bind the cell surface to modulate signaling pathways regulating intracellular trafficking (Soh et al., 2010). Another explanation for altered extracellular protein profiles in the NCLs could be cell death. However, this seems unlikely because not all lysosomal enzymes are detected in abnormal amounts outside cellular models or in extracellular fluid (e.g. CSF, urine).

The exact functions of NCL-related proteins outside the cell are also not entirely clear. Since all secreted NCL-related proteins, except GRN, function as enzymes inside the cell, they perhaps also function as enzymes outside cells. In support of this hypothesis, previous work in *D. discoideum* detected TPP1 and CTSD activity in conditioned buffer (Huber and Mathavarajah, 2019) (Fig. 3). In addition, several pieces of evidence suggest that CLN5 functions as a glycoside hydrolase outside the cell (Huber and Mathavarajah, 2018a,b; McLaren et al., 2021). To confirm these hypotheses, the activities of secreted NCL-related proteins will need to be experimentally validated. By contrast, although it is known that some newly synthesized lysosomal proteins are secreted (Ibata and Yuzaki, 2021), the extracellular presence of NCL-related proteins could indicate a trafficking defect where a mutation causes extracellular trafficking of the protein instead of lysosome localization. Thus, the presence of these proteins outside the cell could serve as a biomarker for the NCLs.

Recent work regarding spinal cord injury and neurodegeneration indicates that enzymes released into the ECM through lysosomal exocytosis degrade the ECM, so that neurons can secrete new building blocks to rebuild synapses (Ibata and Yuzaki, 2021). In addition, stimulating lysosomal exocytosis has been proposed as a therapeutic approach for clearing the cell of accumulated material in lysosomal storage diseases (Tancini et al., 2020). These observations, coupled with the findings summarized in this Review, suggest that mutations in NCL-related genes affect the secretion of material required to rebuild synapses or disturb secretory pathways associated with lysosomal exocytosis, which provides further insight into NCL pathology and potential therapeutic opportunities.

## Conclusions

This Review discussed the extracellular localization of several NCL-related proteins and highlighted findings that link seven of the 13 known NCL-related genes (*PPT1*, *TPP1*, *CLN3*, *DNAJC5*, *CLN5*, *CLN6*, *MFSD8*) to the secretion of other proteins. Work in *D. discoideum* shows that NCL-related gene deficiency affects protein secretion, consequently impacting various cellular and developmental processes during the life cycle. Loss of homologs of NCL-related genes in *D. discoideum* (e.g. *cln3*) also affects the secretion of proteins that are aberrantly secreted in mammalian models of NCL and NCL patients (e.g. CTSB, CTSD, HEXA, HEXB). These findings support the continued use of *D. discoideum* to assess the impact of mutations and altered protein secretion on fundamental cellular and developmental processes. In addition, although the aberrant secretion of proteins in different NCL models is intriguing, recent work also detected aberrant levels of metabolites in CSF obtained from CLN2 disease patients, suggesting that small molecules also serve as effective biomarkers (Sindelar et al., 2018). Finally, accumulated evidence indicates that the unconventional secretion of cellular material is likely to underlie other neurological diseases, including Alzheimer's (i.e. tau protein; Zhang et al., 2021) and Parkinson's disease (i.e. alpha-synuclein; Nakamura et al., 2021), suggesting that protein secretion pathways are viable therapeutic targets for multiple forms of neurodegeneration. Together, this Review highlights the impact of NCL-causing mutations on protein secretion and sets the stage for future studies to explore the roles of NCL-related proteins in secretion, which will provide new therapeutic insights regarding this devastating neurological disease.

## Supplementary Material

Supplementary information
